# Editorial for Special Issue: “Additive Manufacturing Approaches to Produce Drug Delivery Systems”

**DOI:** 10.3390/pharmaceutics14112365

**Published:** 2022-11-02

**Authors:** Joana F. A. Valente, Nuno Alves

**Affiliations:** CDRsp—PL—Centre for Rapid and Sustainable Product Development, Polytechnic of Leiria, 2411-901 Leiria, Portugal

Cutting-edge technologies such as additive manufacturing (AM) have had an enormous impact in a multitude of sectors. The production of drug delivery systems and precise medicine are taken to another level with the application of AM in the pharmaceutical field. Using these super-trend production methodologies, it is possible to quickly produce personalized drug delivery systems that would be impossible to achieve using conventional manufacturing methodologies.

Therefore, the reviews presented in this Special Issue inform readers of the different studies performed in recent years concerning the application of different printing methodologies for drug form production as well as different ink development. As one of the reviews published in this Special Issue, Dr. Billa and Dr. Khalid focus their manuscript on current trends in the application of AM to prepare personalized dosage forms on-demand, concentrating on coupling solid dispersion with fused deposition modelling 3D printing [1]. On the other hand, Soleirol et al. presented the latest findings on selective laser sintering (SLS) 3D printing for the preparation of solid oral forms (SOFs), as well as discussing the opportunities and challenges for this state-of-the-art technology in precision medicine. These authors concluded that the inclusion of “Quality by Design” tools in studies could facilitate the deployment of SLS in clinical practice, particularly where good manufacturing practices (GMPs) for 3D-printing processes are not currently used. Nevertheless, drug stability and powder recycling remain particularly challenging in SLS [2]. Another important thematic approach was bioink development and, in this context, Oliveira et al. (2021) performed a systematic review where different formulations, crosslinking methods, and methods of action, among other topics, were sequentially explored to provide readers with helpful methodological guidelines for the development of novel bioinks [3].

This Special Issue was also furnished with interesting and innovative research papers that could lead to advances in the field of pharmaceutical/drug delivery development and production. A prime example is the study presented by Jung et al. (2021), where high-resolution and high-dimensional microneedles were successfully produced by adjusting the printing angle using a general SLA 3D printer. This technology could be applied to the manufacture of drug delivery tools and various microstructures [4].

Additionally, an array of materials could be conjugated and optimized to accomplish improved drug delivery strategies for different kinds of applications, and these materials/structures must be well-characterized. In this regard, Tesk et al. conducted analyses of the biological, chemical, mechanical and thermal properties of photopolymerized poly(ethylene glycol) diacrylate (PEGDA) and specific copolymers with different photoinitiator concentrations before and after applying a post-treatment washing process. The main goal of this study was to provide readers with information concerning PEGDA multi-materials to contribute to the future development of tools ensuring safe and effective individual therapy for patients [5]. To evaluate the particularities of the printing process, Carabalho and his team studied the extrusion process and physical appearance of filaments made from a hydrophilic polymer and a non-molten model drug. Metformin was used as a model drug and Affinisol™ 15LV as the main carrier. Drug-loaded filaments were obtained by using a single-screw extruder, and their printability was subsequently tested. The findings provided by these authors enabled us to understand the behaviour of filaments containing a non-molten component [6].

Concerning the production of different drug delivery structures using AM, this Special Issue presents an array of research papers focusing on different kinds of structures, different AM production methodologies, and different applications/targets. In this regard, fibres and nanofibres for different purposes were developed in both cases using electrospinning. In the first case, nitric-oxide polymeric fibres were developed to promote an antimicrobial effect [7], and electrospun nanofibres were used as a skin substitute. This skin substitute is based on gelatin and chitosan fibres that not only mimic the skin ECM, but also potentially prevent infections by acting as a drug delivery system for a phlorotannins-enriched extract from Undaria pinnatifida [8].

Another approach to a drug delivery structure could be a scaffold, which was the chosen methodology of Sandri et al. In this study, a medicated osteoinductive and bioresorbable bone graft was designed and investigated for its ability to control antibiotic drug release in situ. This represents an ideal solution to the eradication or prevention of infection, while simultaneously repairing bone defects. Overall, the produced scaffolds were safe and effective as a local delivery systems for an extended duration of therapy. Additionally, promising results for the prevention of bone-defect-related infections in orthopaedic surgeries were reported [9]. Gelinsky and his team also developed scaffolds, but in this case, the main goal was to develop an antibiotic-loaded hydrogel delivery system generated by 3D core/shell extrusion printing that can be flexibly used to produce wound coverage or to be integrated into a tissue substitute. The main findings demonstrated that by changing the composition of the shell hydrogel, the release kinetics can be significantly slowed down and, changing the shell thickness can help to modulate the release of antibiotics [10].

Research concerning the production of oral forms using AM is also presented in this Special Issue. One study focuses on the production of buccal films made of two synthetic polymers, gelatin-polyvinylpyrrolidone and gelatin-poly(vinyl alcohol), and their characterization. The in silico population simulations indicated increased drug bioavailability and decreased inter-individual variations in the resulting pharmacokinetic profiles compared to immediate-release tablets [11]. Saleh et al. produced a self-nanoemulsifying tablet formulation of dapagliflozin propanediol monohydrate using a semisolid pressure-assisted microsyringe (PAM) extrusion-based 3D-printing technique. The formulation system was made of two major components (liquid and solid phase), which include oils and a co-surfactant in the liquid phase. Meanwhile, a surfactant and solid matrix, as solid-phase excipients, ultimately self-nanoemulsify as a drug-encapsulated nanoemulsion system on contact with aqueous phase/gastrointestinal fluid [12].

Counterfeit products in pharmaceutical production is a major subject that is causing great concern. Therefore, a study developed by Quodbach et al. (2021) is presented, where a proposal to solve this problem using AM is made. To test this concept, the authors encoded binary digits (bits) on the surface of fused deposition modelling (FDM) 3D-printed geometries. The most commonly used polymers in pharma were tested, and the scanning and printing processes were evaluated. The main conclusions were that the addition of a colourant or active pharmaceutical ingredient could facilitate this detection process, and the process could also be transferred to 3D-printed pharmaceuticals. However, further improvements are necessary to increase robustness and allow the use of more materials [13].

Moreover, the application of mathematical models in the development of drug delivery systems is also an advantageous approach since, in most cases, it is possible to decrease the costs involved in the drug delivery development process. As an example, Sousa et al. (2021) applied design of experiments (DoE) to quickly and less expensively explore and tailor the characteristics of chitosan/pDNA nanosystems to produce a DNA vaccine [14]. Concerning the vaccine field, Melchels et al. developed elastic bioresorbable polymeric capsules for the osmosis-driven delayed burst delivery of vaccines. This device osmotically delayed delivery and was able to release a payload after a delay of approximately 21 days in a consistent and reproducible manner [15].

Overall, the reports presented in this Special Issue highlight the importance of exploring production approaches based on the use of AM and mathematical models to achieve efficient materials, structures and devices to be applied in the drug delivery field. We, as Guest Editors, are deeply grateful to all the authors for their outstanding-quality research and the critical evaluations of their manuscripts.
